# Supplementary material for: Molecular basis of wax-based color change and UV reflection in dragonflies
Source: eLife. 2019 Jan 15;8:e43045. doi: 10.7554/eLife.43045 (PMC6353593; doi:10.7554/eLife.43045)
Supplement: Supplementary file 1. [file elife-43045-supp1.docx]

Supplementary File 1. *Orthetrum albistylum* samples and RNA sequencing reads.

| Sex | Stage and region^a^ | Collection date^b^ | No. of pairs | HiSeq/MiSeq | Accession no. |
| --- | --- | --- | --- | --- | --- |
| Male  Male  Male  Male  Male  Male  Male  Male  Male  Male  Female  Female  Female  Female  Female  Female  Female  Female  Female  Female  Female  Female | Immature, Dorsal  Immature, Ventral  Semimature, Dorsal  Semimature, Ventral  Mature, Dorsal  Mature, Ventral  Mature-Aged, Dorsal  Mature-Aged, Ventral  Aged, Dorsal  Aged, Ventral  Immature, Dorsal  Immature, Ventral  Semimature, Dorsal  Semimature, Ventral  Mature, Dorsal  Mature, Ventral  Mature-Aged, Dorsal  Mature-Aged, Ventral  Aged, Dorsal  Aged, Ventral  Mature (Androchrome), Dorsal  Mature (Androchrome), Ventral | 07 May 2013  07 May 2013  22 Aug 2011  22 Aug 2011  06 Sep 2012  06 Sep 2012  06 Sep 2012  06 Sep 2012  05 Oct 2015  05 Oct 2015  07 May 2013  07 May 2013  22 Aug 2011  22 Aug 2011  06 Sep 2012  06 Sep 2012  06 Sep 2012  06 Sep 2012  04 Oct 2015  04 Oct 2015  19 Jul 2013  19 Jul 2013 | 11,621,947  14,803,937  7,714,443  14,007,951  15,077,404  14,323,309  14,930,606  15,855,313  1,593,735  1,474,252  14,319,182  12,032,249  7,521,408  10,857,908  15,296,079  14,629,158  11,808,039  16,328,964  1,493,628  1,799,228  8,864,228  18,479,930 | HiSeq 100 bp paired  HiSeq 100 bp paired  HiSeq 100 bp paired  HiSeq 100 bp paired  HiSeq 100 bp paired  HiSeq 100 bp paired  HiSeq 100 bp paired  HiSeq 100 bp paired  MiSeq 300 bp paired  MiSeq 300 bp paired  HiSeq 100 bp paired  HiSeq 100 bp paired  HiSeq 100 bp paired  HiSeq 100 bp paired  HiSeq 100 bp paired  HiSeq 100 bp paired  HiSeq 100 bp paired  HiSeq 100 bp paired  HiSeq 100 bp paired  HiSeq 100 bp paired  MiSeq 300 bp paired  MiSeq 300 bp paired | DRA001709  DRA001710  DRA001687  DRA001690  DRA001697  DRA001698  DRA001700  DRA001701  DRA007015  DRA007016  DRA001712  DRA001713  DRA001693  DRA001694  DRA001703  DRA001704  DRA001706  DRA001707  DRA007017  DRA007018  DRA001716  DRA001717 |

^a^All samples are abdominal epidermis.

^b^All samples were collected by Ryo Futahashi at Tsukuba, Ibaraki, Japan.
